# Changes in Metabolite Patterns During Refrigerated Storage of Lamb's lettuce (*Valerianella locusta* L. Betcke)

**DOI:** 10.3389/fnut.2021.731869

**Published:** 2021-10-06

**Authors:** Valentina Schmitzer, Mateja Senica, Ana Slatnar, Franci Stampar, Jerneja Jakopic

**Affiliations:** ^1^Department of Landscape Architecture, Biotechnical Faculty, University of Ljubljana, Ljubljana, Slovenia; ^2^Department of Agronomy, Biotechnical Faculty, University of Ljubljana, Ljubljana, Slovenia

**Keywords:** *Valerianella locusta*, primary metabolites, phenolics, carotenoids, chlorophyll, storage

## Abstract

Lamb's lettuce is a popular winter salad, often grown in private vegetable plots, small local farms or in intensive vegetable production. It is usually marketed as a ready-to-eat produce in supermarkets. The aim of the study was to evaluate the changes in biochemical composition and degradation of bioactive compounds during consumer-relevant time of home-grown and store-bought *Valerianella locusta* “Vit” salad. Primary metabolites, assimilatory pigments as well as secondary metabolites were monitored during 1 week of refrigerated storage. Home-grown lamb's lettuce exhibited highest levels of total sugars, total organic acids, vitamin C, and total phenolic content as well as enhanced levels of most individual phenolic compounds and chloroplast pigments. Locally produced samples of lamb's lettuce also contained high levels of analyzed bioactive components. All samples retained most bioactive components during the entire period of refrigerated storage. The results underline the instability of vitamin C during refrigerated storage of lamb's lettuce and pinpoint this parameter as being the most affected by storage.

## Introduction

*Valerianella locusta* L. Betcke or lamb's lettuce is a common minimally processed ready-to-eat vegetable. It can be used either as a leafy salad, as an ingredient in salad mixes or in more complex culinary dishes (1, 2). Traditionally, this appealingly termed vegetable was sown in every kitchen garden or in small local greenhouses and consumed during the colder period of the year. It is extremely tolerant to low temperatures and can even survive under snow cover. Now, increased market demand throughout the year has encouraged producers to grow lamb's lettuce on a larger scale, either in greenhouse cultivation or on open fields, and sell their produce to supermarkets and restaurant supply chains.

*Valerianella locusta* is known for its balanced mineral and chemical composition resulting in high nutritional value and favorable taste qualities (3). The rosettes contain many bioactive compounds, such as vitamin C, carotenoids, phenols, folic acid, sterols and omega-3 fatty acids similar to other salads (4–7). Długosz-Grochowska et al. (8) performed a detailed compositional study on lamb's lettuce and reported the presence of three folate forms and seven phenolic compounds. Phenolic acids are the prevalent group of secondary metabolites in *V. locusta*, followed by flavonoid glycosides and free flavonoids (9). Above all, its leaves are one of the richest sources of chlorogenic acid among leafy vegetables and contain very high tetrahydrofolate (also known as vitamin B9) (8) and carotenoid levels (4). During storage bioactive compounds undergo a different rate of degradation and therefore it is preferable that lamb's lettuce, as most leafy salads, is consumed within a week after harvest (9, 10).

Interest for phytonutrient-rich vegetables has increased during the last decades and studies point out that consumers are more and more aware of the benefits of a balanced diet (11). But are pre-packed and ready-to-eat alternatives to home-grown salads really offering the consumer the same portion of health-beneficial compounds? Are the products advertised in supermarkets as locally grown superior to products transported from greenhouses or fields further away? And how do the internal parameters of these products change during storage? Lamb's lettuce (*V. locusta*) “Vit” was chosen as a model plant as it is rich in phenolic compounds, highly perishable and readily available year-round. Moreover, a steady increase of consumer demand for this pre-packed produce can be registered in Europe (12) and on the other hand, its pest free and easy-to-grow characteristics make it one of the most popular salads to grow in private vegetable plots. Home food gardening has received a boost in the last years with more and more people cultivating fresh food (13).

To elucidate the turnover of biochemical compounds in supermarket-bought and home-grown lamb's lettuce this study focused on (1) determining internal quality parameters of different sources of “Vit” lamb's lettuce, and (2) detecting the stability of metabolites during domestic refrigerated storage of lamb's lettuce. A detailed HPLC and HPLC-MS supported analysis of primary and secondary profiles was performed for the period of 1 week to simulate the common consumer's practice of storing lamb's lettuce in sealed plastic bags in household refrigerators. We hypothesized that home-grown samples and locally produced lamb's lettuce are characterized by superior biochemical composition to that of supermarket-bought samples of non-local origin.

## Materials and Methods

### Plant Material

Several bags (4 per treatment, each containing 0.125 kg) of ready-to-eat lamb's lettuce (*V. locusta*) were purchased at three different supermarkets in Ljubljana, Slovenia, at the first day of their commercial life (T0), 24 h after packaging on March 26th 2018 (treatments SM1, SM2, and SM3). All supermarket-bought samples had the same expiry date, were field-grown in an organic production and one of them (SM1) was advertised as a locally grown product. On the same day (March 26th 2018), organic home-grown (HG) lamb's lettuce was collected at a private garden in Lendava, Slovenia (46.56°N, 16.45°E) and transferred immediately to the laboratory facility at Biotechnical faculty in Ljubljana. The cultivation practice for home-grown lamb's lettuce followed the same technological practices applied in large-scale organic vegetable production. All samples were a dark green short leaf “Vit” commercial variety preferred by European consumers and often cultivated on a garden scale. Each treatment was combined into a single batch containing 0.500 kg lamb's lettuce. Prior to analyses, the rosettes were washed in distilled water and gently tapped dry with tissue paper. A portion of each treatment/batch was subjected to immediate analyses on day 1 (T0). To mimic common domestic use, the rest of the batch was stored in plastic bags (4 L) with a zipper, deposited in a refrigerator at 4°C and reopened and analyzed after 2 days (T2), 4 days (T4), and 7 days (T7). On each sampling date, five individual portions (each containing seven rosettes) were ground with liquid nitrogen and all chemical analyses were performed on this source material (*n* = 5).

### Extraction and Determination of Sugars and Organic Acids

Rosettes (1.5 g) were homogenized in 2 ml of double distilled water with an Ultra-Turrax T-25 (Ika-Labortechnik). Extraction was performed at room temperature with constant stirring for 30 min (14). Samples were centrifuged (Eppendorf Centrifuge 5810 R) at 10,621 × g for 10 min at 10°C and the supernatant was filtered through 0.20 μm cellulose ester filters (Macherey-Nagel, Germany) into vials. Five repetitions were carried out (*n* = 5) per treatment and sampling; each repetition consisted of several rosettes. Primary metabolites were analyzed on a high-performance liquid chromatography (HPLC) system (Thermo Separation Products, San Jose, CA, USA) with injection volume of 20 μl and flow rate maintained at 0.6 ml min^−1^. Sugar separation was carried out on a Rezex RCM-monosaccharide column from Phenomenex (Ca+ 2%) at 65°C (300 × 7.8 mm). Double distilled water was used as a mobile phase, total run time was 30 min, and a refractive index (RI) detector was used for carbohydrate detection as described by Weber et al. (15). Analyses of organic acids were performed with a UV detector at 210 nm and 65°C with total run time of 30 min and flow rate of 0.6 ml min^−1^. A Rezex ROA-organic acid [H+ (8%)] column from Phenomenex (300 × 7.8 mm) was used as described by Mikulic-Petkovsek et al. (14) and Weber et al. (15). The elution solvent was 4 mM sulfuric acid in double distilled water. A PDA detector was used to monitor the eluted organic acids.

Corresponding external standard curves were used for quantification of primary metabolites and all compounds were expressed in mg g^−1^ fresh weight (FW). Total sugar content, total organic acids content and sugar/organic acid ratio were calculated as sums (or ratios) of all detected sugars or organic acids.

### Extraction and Determination of Vitamin C

Vitamin C was determined by separately detecting the amount of L-ascorbic acid (AA) and L-dehydroascorbic acid (DHA) according to the method described by Helland et al. (16), with some modifications. Fresh leaves of lamb's lettuce were ground to a powder using liquid nitrogen. For the analysis of AA 1.5 g of leaf powder was extracted with 2 ml of ice-cold 6% *m*-phosphoric acid and for the analysis of DHA with 2 ml of ice-cold 6% *m*-phosphoric acid containing 4 mM EDTA. Extraction was performed with homogenisation on a shaker for 30 s.

A similar HPLC method as for other organic acids was utilized for the quantification of ascorbic. However, it was operated at room temperature and at a different wavelength (245 nm). The identification and quantification were achieved with comparison of the retention time and corresponding external standard curves.

DHA was calculated by subtracting the ascorbic acid content in reduced extract from that of the non-reduced extract. Vitamin C content was defined as the sum of AA and DHA contents.

### Extraction of Phenolic Compounds

Five repetitions were carried out (*n* = 5) per treatment and sampling date; each repetition included several rosettes. Rosettes (1.5 g) were chopped and extracted with 2 ml methanol containing 3% (v/v) formic acid in an iced ultrasonic bath for 1 h as described by Mikulic-Petkovsek et al. (14) and Weber et al. (15). Then, the extracts were centrifuged at 10,621 × g at 4°C for 10 min, filtered through 0.2 μm Chromafil AO-20/25 polyamide filters (Macherey-Nagel, Germany) and transferred to vials. Identical samples were used for determination of total phenolic content.

### Determination of Individual Phenolic Compounds Using HPLC-DAD-MS^n^ Analysis

Thermo Finnigan Accela HPLC system (Thermo Scientific, San Jose, USA) coupled with mass spectrometer with a diode array detector at 280 nm (derivatives of hydroxycinnamic acids) or 350 nm (flavones, flavonols, and flavanone) were used for identification of individual phenolics as described by Weber et al. (15). Spectra were recorded between 200 and 600 nm. A 150 × 4.6 mm i.d., 3 μm, Gemini C_18_ (Phenomenex, Torrance, CA, USA) column operated at 25°C was used. The elution solvents were (A) 3% acetonitrile with 0.1% formic acid in double distilled water (v/v/v) and (B) 3% double distilled water with 0.1% formic acid in acetonitrile (v/v/v). Samples were eluted according to the linear gradient described by Weber et al. (15). The injection amount was 20 μl and the flow rate maintained at 0.6 ml min^−1^.

Phenolics were identified on a mass spectrometer with electrospray ionization (ESI) operating in negative ion mode using full scan data-dependent MS^n^ scanning from *m/z* 115 to 1,500. Operating conditions were previously reported by Weber et al. (15). Spectrometric data were elaborated using the Excalibur software (Thermo Scientific) and retention times, UV spectra and MS^n^ fragmentation were used for compound identification (SM, Table 1). Quantification was achieved by comparing peak areas of the sample and corresponding standard curves. Compounds were expressed in mg kg^−1^ fresh weight (FW). For compounds lacking standards, quantitation was carried out using comparable compounds. Therefore, luteolin-pentosylhexoside, kaempferol-3-*O*-rutinoside, genistin, hesperidin and diosmetin were quantitated in equivalents of luteolin-4'-*O*-glucoside.

**Table 1:** Individual sugars, organic acids, and vitamin C levels (mg kg^−1^ FW ± SE) of different *V. locusta* “Vit” samples prior to refrigerated storage (T0).

	**Sample source^a^**				
**Compound**	**HG**	**SM 1**	**SM 2**	**SM 3**	**Significance**
Fructose	2091.0 ± 108.1^a^	735.4 ± 100.2^b^	224.9 ± 7.5^c^	267.7 ± 15.2^c^	^***^
Glucose	5227.5 ± 270.2^a^	1838.4 ± 250.5^b^	562.2 ± 18.8^c^	669.4 ± 38.1^c^	^***^
Sucrose	59.7 ± 6.3^b^	214.4 ± 81.9^a^	82.0 ± 4.4^b^	280.8 ± 23.6^a^	^***^
Citric acid	1746.9 ± 298.0^a^	1399.9 ± 208.2^a^^b^	1289.0 ± 74.8^a^^b^	1199.9 ± 76.4^b^	^**^
Fumaric acid	66.6 ± 4.0^a^	50.0 ± 7.5^a^	37.3 ± 2.1^b^	33.1 ± 1.2^b^	^***^
Malic acid	5721.8 ± 219.0^a^	5056.3 ± 695.9^a^	3409.4 ± 145.1^b^	3161.4 ± 150.4^b^	^***^
Oxalic acid	63.0 ± 3.8	64.5 ± 9.1	63.0 ± 3.8	58.0 ± 2.8	NS
Quinic acid	1701.2 ± 109.6^a^	1226.1 ± 210.1^b^	776.8 ± 42.8^c^	795.0 ± 48.9^c^	^***^
Shikimic acid	6.5 ± 0.2^a^	5.2 ± 0.7^a^	3.7 ± 0.2^b^	3.7 ± 0.4^b^	^***^
Tartaric acid	232.9 ± 49.7^a^	194.0 ± 43.4^b^	226.1 ± 11.3^a^	207.4 ± 9.2^a^^b^	^***^
Vitamin C	406.6 ± 54.1^a^	239.8 ± 62.8^a^^b^	214.2 ± 24.2^b^	116.9 ± 26.8^b^	^***^

a *Sample source: HG, home-grown sample; SM1, locally-grown supermarket-bought sample; SM2, supermarket-bought sample; SM3, supermarket-bought sample*.

Different letters (a-c) denote statistically significant differences in each compound among V. locusta “Vit” sample sources by Duncan's multiple range test at ***p < 0.001, **p < 0.01, and NS, non-significant. Standard error (SE) following each mean represents the standard deviation of sampling distribution (n = 5).

### Total Phenolic Content

Total phenolic content (TPC) was determined using Folin-Ciocalteau (FC) reagent (five replicates per sampling date) as described by Mikulic-Petkovsek et al. (17). Gallic acid was used as standard and TPC was expressed in mg of gallic acid equivalents (GAE) per kg of fresh weight (FW).

### Extraction and Determination of Chloroplast Pigments

The extraction of chloroplast pigments from lamb's lettuce (1.5 g) was performed with 2 ml ice-cold acetone under dimmed light as described by Sircelj and Batic (18). The extracts were filtered through 0.2 μm polyamide filters and immediately analyzed on the HPLC system.

Analysis of individual carotenoids was carried out on a HPLC system with a DAD detector at 450 nm. Separation of samples was achieved on a Gemini C_18_ column at 25°C and flow rate was maintained at 1 ml min^−1^. The gradient was as follows: from 10 to 70% B in the first 18 min, then linearly to 70% B to 22 min and returning to the initial conditions to the end of the run. Mobile phase A was acetonitrile, double distilled water and methanol (100/10/5; v/v/v) and mobile phase B was acetone with ethyl acetate (2/1; v/v).

Individual carotenoids were further determined on a TSQ Quantum Access Max quadrupole mass spectrometer as previously described by Senica et al. (19). The chromatographic conditions were the same as described above. The MS instrument was operated using an atmospheric pressure chemical ionization (APCI) source in positive ion mode. The APCI parameters were as follows: vaporizer temperature 450°C, capillary temperature 320°C, corona voltage 4.0 kV, sheat gas 55 L/h, auxiliary gas 10 L/h. Mass spectra were scanned in range from *m/z* 70 to 650 and argon was used as collision gas. Data acquisition was performed using Xcalibur 2.2 software. Identification was achieved with mass spectra scans, fragmentation, retention times and spectral properties of target compounds (SM, Table 2).

**Table 2:** Individual phenolic compounds (mg kg^−1^ FW ± SE) in different *V. locusta* “Vit” samples during refrigerated storage.

		**3-CQA^a^**	**5-CQA**	**4-CQA**	**Q-rut**	**Lut-pentosylhex**	**K-rut**	**Genistin**	**Hesperidin**	**Diosmetin**	**Di CQA hex**	**CQA hex**
T0^b^	HG^c^	7.53 ± 0.74^c^	1043.88 ± 39.80^a^	56.23 ± 2.02^a^	11.02 ± 0.37^a^	36.85 ± 1.59^a^	196.14 ± 9.97^a^	10.32 ± 0.33^a^	34.68 ± 1.18^b^	18.89 ± 1.39^b^	102.53 ± 3.29^b^	0.95 ± 0.16^d^
	SM 1	8.12 ± 0.99^b^^c^	831.22 ± 48.45^b^	46.81 ± 2.69^b^	7.96 ± 0.33^b^	19.88 ± 0.91^b^	67.42 ± 5.07^b^	5.65 ± 0.37^b^	50.99 ± 0.83^a^	25.77 ± 0.73^c^	146.69 ± 11.37^a^	9.11 ± 0.58^a^
	SM 2	10.75 ± 0.86^a^^b^	423.42 ± 33.14^c^	21.14 ± 1.15^c^	4.67 ± 0.27^c^	5.86 ± 0.41^d^	18.33 ± 1.61^c^	2.02 ± 0.15^c^	21.92 ± 1.12^c^	10.98 ± 0.51^b^^c^	54.06 ± 3.73^c^	3.11 ± 0.28^c^
	SM 3	13.76 ± 1.51^c^	730.15 ± 69.84^b^	42.96 ± 3.88^b^	5.38 ± 0.29^c^	9.05 ± 0.90^c^	27.77 ± 2.66^c^	1.70 ± 0.22^c^	31.39 ± 3.12^b^	15.27 ± 2.46^b^	97.84 ± 10.71^b^	6.46 ± 0.73^b^
		^***^	^***^	^***^	^***^	^***^	^***^	^***^	^***^	^***^	^***^	^***^
T2	HG	6.70 ± 0.32^c^	1014.05 ± 58.18^a^	56.54 ± 1.79^a^	11.92 ± 0.49^a^	36.85 ± 1.59^a^	214.34 ± 4.55^a^	10.67 ± 0 42^a^	44.77 ± 0.84^a^	19.83 ± 0.49^a^	116.61 ± 29.48^a^^b^	8.18 ± 0.54^b^
	SM 1	10.59 ± 1.72^b^	885.02 ± 222.80^a^^b^	51.53 ± 1.13^b^	9.57 ± 0.35^b^	19.88 ± 0.91^b^	93.35 ± 11.53^b^	5.96 ± 0.26^b^	44.11 ± 1.27^a^	21.79 ±1.10^a^	159.58 ± 10.44^a^	9.38 ± 0.34^a^
	SM 2	9.12 ± 0.37^b^^c^	453.19 ± 33.14^c^	24.72 ± 2.14^d^	4.85 ± 0.27^c^	5.86 ± 0.41^d^	18.55 ± 1.89^c^	2.00 ± 0.14^c^	31.02 ± 2.56^c^	13.06 ± 1.17^c^	79.55 ± 9.42^c^	4.59 ± 0.49^c^^d^
	SM 3	14.26 ± 0.99^a^	639.14 ± 11.77^b^^c^	35.11 ± 1.44^c^	5.37 ± 0.48^c^	9.05 ± 0.90^c^	30.06 ± 0.77^c^	2.53 ± 0.13^c^	38.58 ± 0.28^b^	16.26 ± 0.70^b^	83.41 ± 4.13^b^	5.69 ± 0.23^c^
		^***^	^*^	^***^	^***^	^***^	^**^	^***^	^***^	^***^	^*^	^***^
T4	HG	7.12 ± 0.65	1048.41 ± 41.87^a^	52.84 ± 2.28^a^	12.11 ± 0.85^a^	36.27 ± 2.30^a^	200.88 ± 12.02^a^	10.69 ± 0.68^a^	32.69 ± 0.94^a^	19.22 ± 0.70^a^	149.56 ± 8.82^a^	8.26 ± 0.45a
	SM 1	6.61 ± 0.93	896.28 ± 27.56^b^	44.64 ± 2.28^b^	7.09 ± 0.42^b^	15.45 ± 1.18^b^	72.22 ± 5.52^b^	4.54 ± 0.27^b^	30.41 ± 1.63^a^^b^	18.27 ± 0.90^b^	153.4 ± 7.14^a^	7.77 ± 0.61^a^^b^
	SM 2	13.13 ± 2.96	490.88 ± 38.36^d^	26.65 ± 2.18^c^	5.30 ± 0.32^c^	7.20 ± 1.20^c^	23.63 ± 5.94^c^	2.05 ± 0.34^c^	22.18 ± 1.37^c^	12.93 ± 0.65^b^	82.02 ± 8.15^c^	4.38 ± 0.48^c^
	SM 3	9.64 ± 2.27	753.57 ± 27.74^c^	45.77 ± 1.20^b^	5.43 ± 0.09^c^	9.14 ± 0.78^c^	29.10 ± 1.83^c^	1.92 ± 0.22^c^	27.35 ± 1.39^b^	12.61 ± 3.22^a^	114.99 ± 3.75^b^	6.44 ± 0.37^b^
		NS	^***^	^***^	^***^	^***^	^***^	^***^	^***^	^*^	^***^	^***^
T7	HG	9.29 ± 0.32	1067.30 ±39.52^a^	51.51 ± 1.76^a^	11.00 ± 0.42^a^	32.32 ± 0.92^a^	191.47 ± 8.35^a^	9.05 ± 0.17^a^	31.04 ± 1.62	16.69 ± 0.69	156.56 ± 6.26^a^	8.13 ± 0.70^a^
	SM 1	9.20 ± 0.93	962.78 ± 35.59^b^	48.79 ± 1.00^a^^b^	7.82 ± 0.36^b^	15.36 ± 1.28^b^	62.63 ± 6.10^b^	4.77 ± 0.31^b^	37.00 ± 3.41	18.17 ± 1.76	185.17 ± 7.47^a^	8.73 ± 0.28^a^
	SM 2	12.01± 1.28	456.89 ± 21.58^d^	23.66 ± 0.95^c^	5.22 ± 0.48^c^	6.60 ± 0.55^c^	20.74 ± 0.97^c^	1.84 ± 0.09^c^	23.53 ± 3.44	12.20 ± 1.69	94.25 ± 4.03^c^	5.30 ± 0.87^b^
	SM 3	11.72 ± 0.99	776.57 ± 39.23^c^	43.84 ± 3.04^b^	5.03 ± 0.19^c^	9.14 ± 0.31^c^	31.60 ± 1.21^c^	2.14± 0.18^c^	29.75 ± 1.62	13.94 ± 1.90	109.71 ± 6.22^b^	7.33 ± 0.86^b^^c^
		NS	^***^	^***^	^***^	^***^	^***^	^***^	NS	NS	^***^	^*^

a *Compound identification: 5-CQA, 5-O-caffeoylquinic acid; 3-CQA, 3-O-caffeoylquinic acid; 4-CQA, 4-O-caffeoylquinic acid (cryptochlorogenic acid); Q-rut, quercetin-3-rutinoside; Lut-pentosylhex, luteolin-pentosylhexoside; K-rut, kaempferol-3-O-rutinoside; Di CQA hex, di-caffeoylquinic acid hexoside; CQA hex, caffeoylquinic acid hexoside*.

b *Sampling dates: T0, immediately after purchase or harvest, prior to refrigerated storage; T2, two days in refrigerated storage; T4, four days in refrigerated storage; T7, days in refrigerated storage*.

c *Sample source: HG, home-grown sample; SM1, locally grown supermarket-bought sample; SM2, supermarket-bought sample; SM3, supermarket-bought sample*.

Different letters (a-d) denote statistically significant differences in each individual compound among V. locusta “Vit” sample sources by Duncan's multiple range test at ***p < 0.001, **p < 0.01,*p < 0.05 and NS, non-significant separately for each sampling. Standard error (SE) following each mean represents the standard deviation of sampling distribution (n = 5).

### Chemicals and Products

The following standards were used for determination of sugars and organic acids: sucrose, fructose and glucose, as well as citric, malic, oxalic, tartaric and fumaric, ascorbic acid from Fluka Chemie (Buchs, Switzerland); shikimic and quinic acid from Sigma-Aldrich Chemicals (St. Louis, MO, USA). Standards for phenolic compounds were acquired from Sigma-Aldrich Chemicals (3-caffeoylquinic acid, 4-caffeoylquinic acid, 5-caffeoylquinic acid, luteolin-4'-*O*-glucoside, quercetin-3-*O*-rutinoside).

Zeaxanthin, chlorophyll a and b, β-carotene and lutein were from Sigma-Aldricht Chemie (Steinheim, Germany) and neoxanthin, violaxanthin, antheraxanthin and β-cryptoxanthin from DHI LAB Product (Hørsholm, Denmark). Methanol for the extraction of phenolics was obtained at Sigma-Aldrich Chemicals. The chemicals for the mobile phase were HPLC-MS grade acetonitrile, sulphuric acid and formic acid from Sigma-Aldrich Chemicals. Water for the mobile phase was double distilled and purified with a Milli-Q system (Millipore, Bedford, MA, USA).

### Statistical Analyses

Data were statistically analyzed with the program Statgraphics Centurion XVII (Manugistics, Inc., Rockville, MD, USA) using one-way analysis of variance (ANOVA). Differences between samples, separately for each sampling date, were estimated using the Duncan multiple range test. *P*-values of <0.05 were considered statistically significant.

## Results and Discussion

### Sugars, Organic Acids, and Vitamin C

**Figure 1:**
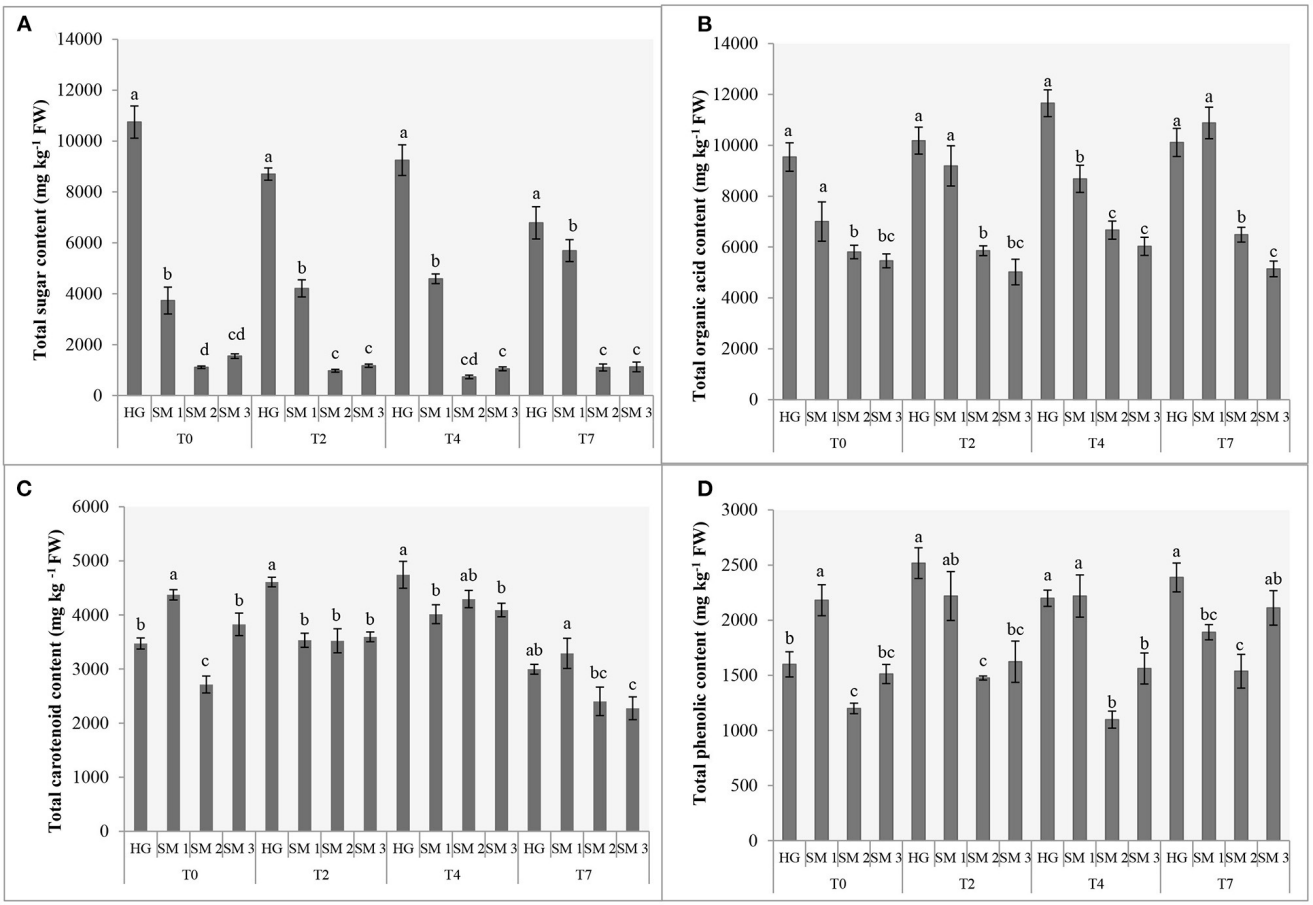
Sums (mg kg^−1^ FW) of sugars **(A)**, organic acids **(B)** and carotenoids **(C)** and total phenolic content **(D)** of different sources of lamb's lettuce subjected to refrigerated storage (T0, prior to storage; T2, 2 days in refrigerated storage; T4, 4 days in refrigerated storage; T7, 7 days in refrigerated storage). Different letters (a-d) above bars denote statistically significant differences in each parameter among *V. locusta* “Vit” sample sources by Duncan's multiple range test at *P* < 0.05 separately for each sampling. Standard errors (SE) are presented with bars within each column.

The content of individual sugars, organic acids and vitamin C in *V. locusta* leaves during 1-week refrigerated storage were studied for the first time. In all samples analyzed in our study glucose was present in highest amounts, followed by fructose and significantly lower levels of sucrose (Table 1). The content of sucrose in lamb's lettuce was comparable to that of baby leaf lettuce reported by Spinardi and Ferrante (20). Similar sugar composition of lamb's lettuce was also described by Enninghorst and Lippert (1), but their research did not report specific contents of these primary metabolites. Recently, sugar levels in lamb's lettuce were analyzed by anthrone colorimetric method, which detects the total amount of soluble sugars in the matrix but fails to specify the content of individual carbohydrates (3, 6). Their results are comparable to the sum of individual sugars, identified in our samples (Figure 1A).

Regardless of the sampling date, HG lamb's lettuce was characterized with up to almost three-fold levels of total sugars (TS) compared to SM1 sample, which was second richest in this group of primary metabolites. Significantly lower levels of TS were recorded in SM2 and SM3 samples and both supermarket-bought samples also demonstrated a comparable sugar turnover during refrigerated storage (Figure 1A). Although an ~36% decrease of TS was detected on the 7th day of HG lamb's lettuce storage, this sample still contained the highest levels of TS compared to other *V. locusta* sources on the last sampling. Comparably, Braidot et al. (4) measured 40% less glucose in lamb's lettuce on the sixth day of refrigerated storage which is consistent with the results on total sugars in our study (Figure 1A). Decreased levels of carbohydrates during the postharvest storage period have directly been linked to lamb's lettuce respiration for which the sugars represent the main substrates (1, 20). Similar findings have been reported on shredded cabbage (21) and turnip (16) and the authors clarified the carbohydrate depletion in samples with increased respiration as well as decomposition correlating with longer storage period. The processes have been extensively studied by McKenzie et al. (22) who monitored ethanolic and lactic fermentation pathway in asparagus and broccoli during storage and linked these with reduced levels of glucose, fructose, and sucrose in different cell compartments.

Extensive analysis of organic acids in various fruits and vegetables, including lamb's lettuce, was undertaken by Flores et al. (23), who detected 10 different compounds in this group of metabolites in fresh *V. locusta* leaves albeit several were only present in traces. All samples analyzed in the present study contained highest amounts of citric and malic acids and lower levels of fumaric, oxalic, quinic, shikimic and tartaric acids (Table 1), which is consistent with the report of Flores et al. (23). Comparable (and highest) levels of total organic acids (TOA) were measured in HG and SM1 samples during the entire period of refrigerated storage. Only on day 4 did we detect significantly higher levels of TOA in HG sample compared to locally grown supermarket-bought lamb's lettuce. Initial levels of TOA were significantly lower in SM2 and SM3 samples, but the time trend of TOA was similar for both sources with an exception on the second sampling. An increase in TOA was recorded on the second and fourth day of storage in most *V. locusta* samples followed by a decrease on day 7 (Figure 1B). TOA pattern (mostly consisting of citric and malic acid in *V. locusta* rosettes) corresponds to the report of Tsuchida et al. (24), who monitored organic acid metabolism during cucumber storage. The authors reported a dramatic increase of malic and citric acid in first 3 days of cold storage, followed by decreased levels of these organic acids at later samplings. The final negative turnover of TOA has been linked to reduced activity of citrate synthase and the role of the enzyme in the Krebs cycle.

Lamb's lettuce is characterized by high ascorbate content compared to other leafy salads (25). The highest levels of vitamin C were detected in HG samples during the entire period of refrigerated storage, followed by SM1 sample (Table 1). Lowest levels of vitamin C were in SM2 and SM3 samples on all sampling dates. The evolution of vitamin C during storage was comparable among all samples and an average of 40% decrease was measured at the end of refrigerated storage. These data are in accordance with the report of Ferrante et al. (25) and Preti and Vinci (26), who detected from 44 to 49% decrease of AA 7 days after *V. locusta* rosettes were stored in a refrigerator. A loss of vitamin C (particularly ascorbic acid) in vegetables during postharvest handling procedures and storage has been reported in other papers, for instance on spinach (27), green beans (28), Japanese radish and green pepper (29) and baby lettuce (20). Vitamin C and specifically, ascorbic acid assimilation in plant tissue is associated with the metabolism of carbohydrates (30) and Wojciechowska et al. (3) confirmed a slight positive correlation between the content of sugars and ascorbic acid in the leaves of lamb's lettuce. A similar correlation was not confirmed for vitamin C in our study. The initial levels of vitamin C in our samples of lamb's lettuce were similar to the results of Wojciechowska et al. (3) as well as Colonna et al. (31). The decrease in ascorbate content is expected during handling and storage of vegetables and has been ascribed to its water solubility, thermic degradation and enzymatic oxidation (32).

### Total Phenolic Content

The content of phenolics was determined with the standard Folin-Ciocalteu assay which revealed significant differences among the sources of lamb's lettuce subjected to refrigerated storage (Figure 1D). Highest TPC (2,181 mg kg^−1^ GAE FW) of fresh samples was determined in SM1 sample, followed by home-grown *V. locusta* rosettes (1,599 mg kg^−1^ GAE FW). The values were in range with previously reported data on lamb's lettuce (6, 8, 33) but significantly lower compared to the TPC reported by Hawrylak-Nowak et al. (34), who measured from 4,000 to 7,000 mg kg^−1^ GAE FW in lamb's lettuce subjected to high temperature stress. Interestingly, TPC increased on the third day of storage in all samples and then either remained constant or increased further. The results are in accordance with the report of Preti and Vinci (26) who measured equivalent content of phenolics in lamb's lettuce during a 7-day refrigerated storage. Similarly, Myojin et al. (29) determined increased TPC of shredded red and white cabbage during a 7-day refrigerated storage and Santos et al. (11) reported a comparable TPC turnover of fresh-cut aromatic herbs. Higher TPC of fresh herbs was tentatively explained with stress-related formation of phenolics following postharvest wounding. Galani et al. (32) linked increased synthesis of phenolic compounds in vegetables under low temperature stress during storage with up-regulated activity of phenylalanine ammonialyase, coupled with low level of polyphenoloxidase activity reducing the oxidation level of phenolic substrates.

### Individual Phenolic Compounds

Twelve phenolic compounds were identified in all samples of lamb's lettuce: five compounds from the group of hydroxycinnamic acids (all derivatives of caffeoylquinic acid), two flavonols (quercetin-3-*O*-rutinoside, kaempferol-3-*O*-rutinoside), two flavones (luteolin-pentosyl hexoside, diosmetin), an isoflavone (genistin) and a flavanone (hesperidin). Hydroxycinnamic acids (particularly 5CQA) represented the most abundant phenolic class in *V. locusta*, regardless of sample origin or duration of refrigerated storage (Table 2). Similarly, Grzegorzewski et al. (9), Ramos-Bueno et al. (5) and Długosz-Grochowska et al. (6) measured highest levels of 5CQA in lamb's lettuce. The levels of this compound exceeded 1,000 mg kg^−1^ FW in some of our samples, which is consistent with the report of Długosz-Grochowska et al. (8). Other phenolic acids, specifically 3CQA and 4CQA were present in much smaller amounts (Table 2) and have been reported in lamb's lettuce for the first time. The levels of rutin and diosmetin were in range with the reports of Długosz-Grochowska et al. (6) but, contrary, higher levels of hesperidin were detected in our samples.

The highest content of 5CQA was detected in HG samples regardless of sampling date. Levels of hydroxycinnamic acids remained constant or even increased during refrigerated storage of lamb's lettuce. Interestingly, comparable content of most individual phenolic compounds was detected in *V. locusta* samples during refrigerated storage. For instance, the content of kaempferol-3-*O*-rutinoside increased in all samples during the first 4 days in the refrigerator which is in accordance with the study of DuPont et al. (35). The authors evaluated compositional changes of lettuce and endive during cold storage and detected a net gain of the prevalent form of kaempferol and explained it with a release of this component from an unidentified precursor. A similar pattern was also reported by Santos et al. (11), who studied phenolic turnover in fresh-cut aromatic herbs. An increase in phenolic compounds during cold storage of *V. locusta* samples may be explained by the activation of phenylalanine ammonia lyase—a key enzyme in biosynthetic pathway, which is triggered by cold-stress conditions (36). Ferrante et al. (25) also proposed that the increase of phenols may counteract the loss of ascorbic acid and balance the shift in antioxidant status of stored rosettes.

### Chloroplast Pigments

Two groups of chloroplast pigments were determined in *V. locusta* samples, namely chlorophylls and carotenoids. Chlorophyll a was the prevalent form of chlorophyll pigments in all samples followed by chlorophyll b, regardless of storage days (Table 3). Home-grown sample initially contained highest levels of total chlorophyll but during refrigerated storage this trait was no longer consistent. Total chlorophyll content was in accordance with previously reported data on lamb's lettuce (25, 34). In addition to chlorophyll a and b, pheophytin was also detected in *V. locusta* samples like in the study of Braidot et al. (4). The molecule is structurally analogous to chlorophyll but lacks the central Mg^2+^ ion and is often present as a degradation product of chlorophyll a (37).

**Table 3:** Individual carotenoids (mg kg^−1^ FW ± SE) in different *V. locusta* “Vit” samples during refrigerated storage.

**Date**		**Antheraxanthin**	**β-cryptoxanthin**	**β-carotene**	**Chlorophyll a**	**Chlorophyll b**	**Lutein**	**Neoxanthin**	**Pheophytin**	**Violaxanthin**	**Zeaxanthin**
T0^a^	HG^b^	140.05 ± 11.41	8.02 ± 0.81.	7.90 ± 0.89^a^	2030.84 ± 330.16^a^	909.02 ± 46.57^a^	1381.51 ± 174.30^b^	2163.89 ± 89.33	26.67 ± 5.66	130.15 ± 12.30^b^	40.71 ± 3.59^b^
	SM 1	168.89 ± 19.02	13.04 ± 1.65	1.15 ± 0.06^b^	783.90 ± 175.21^b^	624.28 ± 21.13^a^	1926.34 ± 146.99^a^	2212.23 ± 67.29	35.36 ± 3.11	183.30 ± 3.45^b^	67.15 ± 3.00^a^
	SM 2	117.88 ± 17.78	10.59 ± 2.59	1.85 ± 0.07^b^	1003.37 ± 445.19^b^	567.45 ± 67.80^a^	1500.36 ± 87.93^a^^b^	1870.31 ± 61.36	32.00 ± 1.04	142.82 ± 5.91^b^	70.60 ± 7.01^a^
	SM 3	161.53 ± 2.27	16.26 ± 0.40	0.70 ± 0.03^b^	419.82 ± 13.85^b^	144.41 ± 6.31^b^	1538.50 ± 14.86^a^^b^	1742.74 ± 71.99	15.23 ± 1.96	288.42 ± 15.49^a^	78.42 ± 1.49^a^
		NS	NS	^*^	^**^	^**^	^**^	NS	NS	^**^	^**^
T2	HG	93.16 ± 5.88	5.28 ± 0.12^a^^b^	2.00 ± 0.24	1426.02 ± 174.02^b^	771.43 ± 62.05^b^	1432.81 ± 51.50	2600.80 ± 72.68^a^	11.49 ± 2.22	62.22 ± 2.14^b^	52.24 ± 8.27
	SM 1	62.16 ± 2.18	1.91 ± 0.23^c^	4.53 ± 0.15	2288.26 ± 408.07^a^^b^	1148.67 ± 42.30^a^^b^	1436.76 ± 89.59	1741.13 ± 82.88^b^	16.63 ± 3.43	37.52 ± 2.37^b^	48.07 ± 2.48
	SM 2	70.22 ± 6.11	7.09 ± 0.69^a^	2.59 ± 0.18	3307.13 ± 722.86^a^	1467.46 ± 98.91^a^	1561.34 ± 91.54	1966.82 ± 52.52^b^	10.90 ± 4.16	54.23 ± 2.62^b^	60.26 ± 5.79
	SM 3	79.19 ± 5.00	4.24 ± 0.29^b^^c^	1.33 ± 0.45	1456.28 ± 233.91^b^	881.28 ± 62.05^b^	1472.29 ± 32.11	1903.96 ± 60.96^b^	7.33 ± 0.70	81.37 ± 4.94^a^	52.48 ± 2.40
		NS	^**^	NS	^*^	^*^	NS	^**^	NS	^*^	NS
T4	HG	80.68 ± 9.40^b^	6.61 ± 0.14b^c^	1.76 ± 0.16^a^	4424.45 ± 317.35^a^	1673.16 ± 16.59^a^	1991.62 ± 171.70^a^	2369.35 ± 164.82^a^	26.79 ± 3.90	37.14 ± 2.45^d^	39.51 ± 1.47^b^
	SM 1	72.48 ± 3.69^b^	5.42 ± 0.15^c^	0.39 ± 0.02^b^	1866.76 ± 151.29^b^	996.66 ± 51.36^c^	1519.61 ± 95.63^b^	1874.11 ± 87.54^b^	16.97 ± 1.21	61.68 ± 4.85^c^	80.40 ± 3.16^a^
	SM 2	100.03 ± 4.00^a^	10.15 ± 1.31^a^^b^	0.36 ± 0.05^b^	2231.11 ± 290.70^b^	1035.72 ± 15.86^b^	1884.56 ± 43.72^a^	2142.18 ± 118.28^a^^b^	24.07 ± 0.43	82.44 ± 6.31^b^	74.04 ± 5.30^a^
	SM 3	108.22 ± 2.92^a^	12.17 ± 1.48^a^	0.83 ± 0.20^b^	1167.61 ± 94.10^c^	892.33 ± 59.52^c^	1800.35 ± 81.73^a^^b^	1950.77 ± 50.44^b^	16.98 ± 2.38	134.78 ± 4.93^a^	83.33 ± 2.78^a^
		^**^	^**^	^**^	^***^	^***^	^*^	^*^	NS	^***^	^***^
T7	HG	36.67 ± 2.10	0.63 ± 0.06^a^^b^	3.83 ± 0.17	1454.77 ± 248.90	690.36 ± 34.52	1151.34 ± 48.87	1712.05 ± 61.67	7.98 ± 0.12^a^	41.30 ± 3.43^b^	50.07 ± 4.78
	SM 1	32.94 ± 0.99	1.10 ± 0.02^b^	4.91 ± 0.29	1197.10 ± 182.05	521.66 ± 25.72	837.81 ± 37.88	1461.33 ± 118.02	4.12 ± 0.02^b^	91.74 ± 6.31^a^	59.99 ± 2.91
	SM 2	33.11 ± 2.73	0.56 ± 0.11^b^	1.73 ± 0.04	523.58 ± 109.42	299.29 ± 17.72	793.88 ± 28.28	1446.72 ± 107.12	1.91 ± 0.05^b^	57.81 ± 3.36^a^^b^	69.15 ± 7.18
	SM 3	43.82 ± 3.86	1.41 ± 0.02^a^	4.26 ± 1.23	905.54 ± 179.51	498.70 ± 25.58	999.40 ± 37.52	1443.18 ± 94.61	3.51 ± 0.01^b^	100.94 ± 9.87^a^	82.09 ± 4.14
		NS	^**^	NS	NS	NS	NS	NS	^*^	^*^	NS

a *Sampling dates: T0, immediately after purchase or harvest, prior to refrigerated storage; T2, two days in refrigerated storage; T4, four days in refrigerated storage; T7, days in refrigerated storage*.

b *Sample source: HG, home-grown sample; SM1, locally-grown supermarket-bought sample; SM2, supermarket-bought sample; SM3, supermarket-bought sample*.

Different letters (a-d) denote statistically significant differences in each individual compound among V. locusta “Vit” sample sources by Duncan's multiple range test at ***p < 0.001, **p < 0.01,*p < 0.05 and NS, non-significant separately for each sampling. Standard error (SE) following each mean represents the standard deviation of sampling distribution (n = 5).

The content of chlorophylls in most *V. locusta* samples was significantly reduced only after 7 days of refrigerated storage (Table 3). Correspondingly, Braidot et al. (4) detected deleterious effects of storage on pigment contents of *V. locusta* samples after at least 5 days of storage. A different methodological approach in evaluating chlorophyll turnover was undertaken by Manzocco et al. (38), who measured decreased SPAD index on approx. 10th day in refrigerated storage. The reduction of chlorophyll content during cold storage of *V. locusta* seems to be very slow and depends on the duration and storage parameters (25, 39). Similar results were reported for Swiss chard, rocket and baby lettuce (20, 25, 40).

Carotenoids are potent antioxidant molecules and among them lutein is frequently found in leafy vegetables (5). In addition to this carotenoid, six other forms of assimilation pigments were confirmed in *V. locusta* samples: antheraxanthin, β-cryptoxanthin, β-carotene, neoxanthin, violaxanthin and zeaxanthin. Carotenoid identification was in accordance with the study of Ramos-Bueno et al. (5) except for antheraxanthin, which has been determined in lamb's lettuce for the first time. Neoxanthin was the most abundant carotenoid in all samples, followed by lutein (Table 3). Similar carotenoid composition and content were reported by Długosz-Grochowska et al. (6) apart from higher levels of β-carotene in their study. However, the transitory nature of this carotenoid may explain higher levels of violaxanthin and zeaxanthin in our samples as β-carotene is an important intermediate in formation of xanthophylls (41). Generally, the highest sum of carotenoids was monitored in home-grown and locally grown rosettes, but the significance was not detected on all samplings (Figure 1C). Carotenoids proved similarly stable during the first 5 days of storage compared to chlorophyll pigments, but a decrease after this initial period has been detected in our samples. Consistently, Ferrante et al. (25) and Spinardi and Ferrante (20) reported the reduction of carotenoid content in *V. locusta* rosettes only after 8 days of storage at 4°C or even 10°C but Kołton et al. (33) failed to report any changes in total carotenoids even after 3 weeks of storage.

## Conclusions

Home-grown lamb's lettuce is generally hand-picked in the garden, cleaned and consumed within the same day. According to UNECE standard for Lamb's lettuce (42), sealed bags of fresh and turgid rosettes should also be dispatched to the supermarket as fast as possible; preferably on the day of harvest. However, the sequence of procedures necessary to produce ready-to-eat product (i.e., washing, removing root tufts or cotyledons, packaging and sealing) and transport to the supermarket shelf promotes the biochemical instability of the product itself (12). A loss of primary and secondary metabolites was expected in all samples after harvest, but particularly in those stored for a longer period of time (i.e., all SM samples). Results underline the instability of vitamin C during refrigerated storage of lamb's lettuce and pinpoint this parameter as being the most affected by storage. Other primary and secondary metabolites were less affected by storage but as a rule, home-grown samples (or in some cases locally produced supermarket-bought lamb's lettuce) demonstrated highest levels of most compounds on all samplings. These samples also retained most bioactive components after a 7-day storage which confirms the superiority of local and home-grown produce compared to other foodstuffs for the entire period of consumer-relevant time.

## Data Availability Statement

The original contributions presented in the study are included in the article/Supplementary Material, further inquiries can be directed to the corresponding author/s.

## Author Contributions

FS provided an initial idea, got in touch with growers and suppliers to ensure Vit samples were all grown organically according to the same measures. AS was in charge of sampling, coordinated laboratory work, and compound identification. JJ was in charge of compound identification and optimization of analytical methods. MS analyzed the samples in the laboratory and did statistical analysis. VS wrote the manuscript. All authors contributed to the manuscript.

## Funding

This research was a part of program Horticulture No. P4-0013-0481 funded by the Slovenian Research Agency (ARRS).

## Conflict of Interest

The authors declare that the research was conducted in the absence of any commercial or financial relationships that could be construed as a potential conflict of interest.

## Publisher's Note

All claims expressed in this article are solely those of the authors and do not necessarily represent those of their affiliated organizations, or those of the publisher, the editors and the reviewers. Any product that may be evaluated in this article, or claim that may be made by its manufacturer, is not guaranteed or endorsed by the publisher.
