# Supplementary material for: Changes in Metabolite Patterns During Refrigerated Storage of Lamb's lettuce (Valerianella locusta L. Betcke)
Source: Front Nutr. 2021 Oct 6;8:731869. doi: 10.3389/fnut.2021.731869 (PMC8526726; doi:10.3389/fnut.2021.731869)
Supplement: Supplementary file 1 [file Table_1.DOCX]

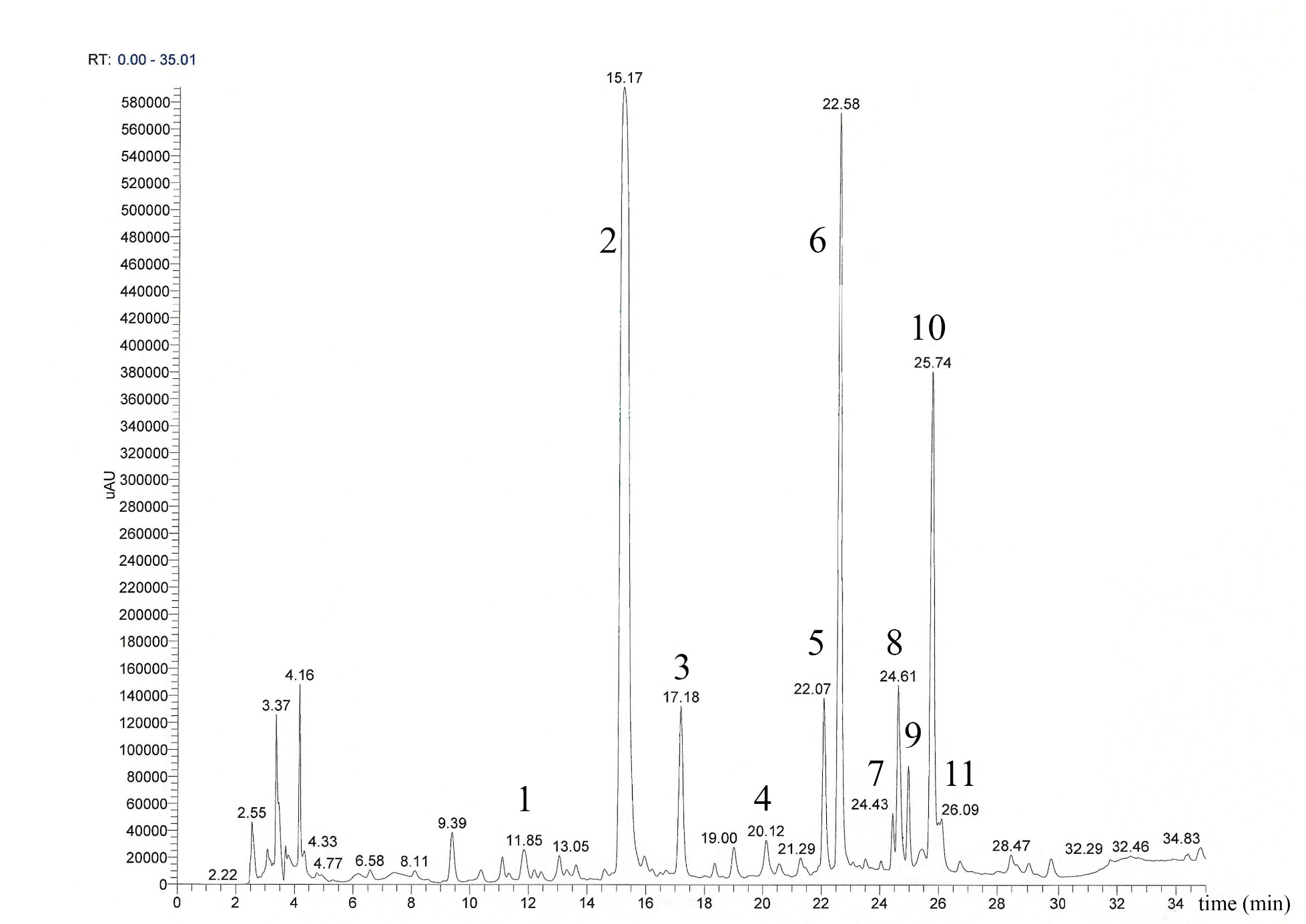


| No. | Compound acronym | Phenolic compound | Rt (min) | [M−H]^−^ (m/z) | MS/MS (m/z) |
| --- | --- | --- | --- | --- | --- |
| 1 | 3-CQA | 3-*O*-caffeoylquinic acid | 11,85 | 353 | 191, 179, 135 |
| 2 | 5-CQA | 5-*O*-caffeoylquinic acid | 15,17 | 353 | 191 |
| 3 | 4-CQA | 4-*O*-caffeoylquinic acid (cryptochlorogenic acid) | 17,18 | 353 | 191 |
| 4 | Q-rut | quercetin-3-rutinoside | 20,12 | 609 | 301 |
| 5 | Lut-pentosylhex | luteolin-pentosylhexoside | 22,07 | 579 | 285,199, 217, 241,133 |
| 6 | K-rut | kaempferol-3-*O*-rutinoside | 22,58 | 285 | 199, 241, 257 |
| 7 | Genistin | Genistin | 24,43 | 431 | 269 |
| 8 | Hesperidin | Hesperidin | 24,61 | 609 | 301 |
| 9 | Diosmetin | Diosmetin | 24,61 | 399 | 299, 284) |
| 10 | Di CQA hex | di-caffeoylquinic acid hexoside | 25,74 | 516 | 353,191,179,135 |
| 11 | CQA hex | caffeoylquinic acid hexoside | 29,09 | 516 | 353,191 |

A representative chromatographic profile of identified phenolic compounds in Lamb's lettuce with [M−H]^−^ (m/z) and MS/MS (m/z) fragmentation data.
