# Supplementary figures and images for: Changes in Metabolite Patterns During Refrigerated Storage of Lamb's lettuce (Valerianella locusta L. Betcke)
Source: Front Nutr. 2021 Oct 6;8:731869. doi: 10.3389/fnut.2021.731869 (PMC8526726; doi:10.3389/fnut.2021.731869)

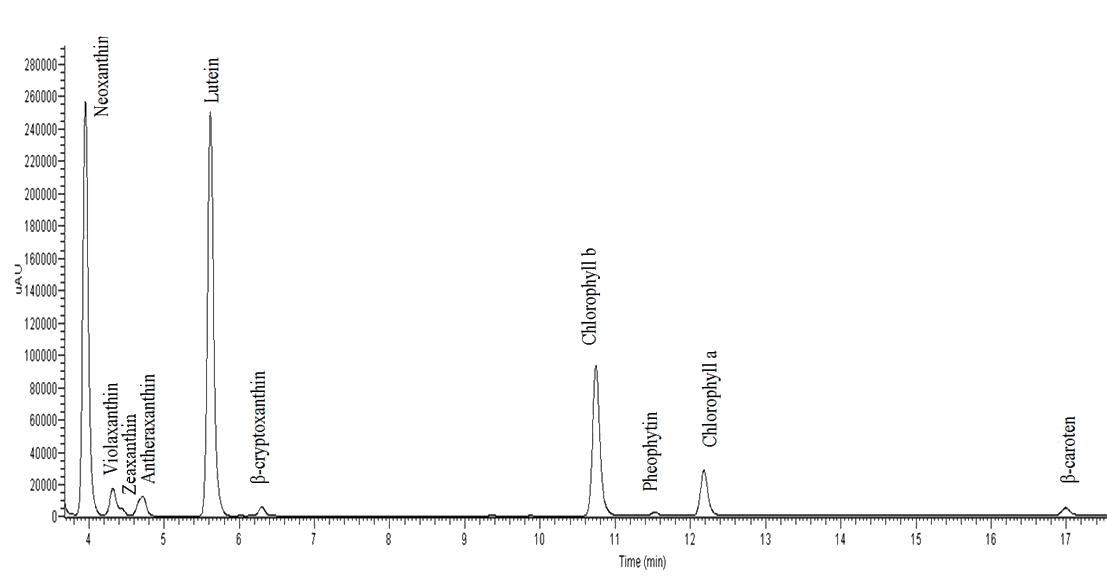


A representative chromatographic profile of identified carotenoids in Lamb's lettuce.

Supplement: Supplementary file 2 [file Table_2.DOCX]
